# Cerebellar deep brain stimulation as a dual-function therapeutic for restoring movement and sleep in dystonic mice

**DOI:** 10.1016/j.neurot.2024.e00467

**Published:** 2024-10-23

**Authors:** Luis E. Salazar Leon, Linda H. Kim, Roy V. Sillitoe

**Affiliations:** a Department of Neuroscience, Baylor College of Medicine, Houston, TX, USA; b Department of Pathology & Immunology, Baylor College of Medicine, Houston, TX, USA; c Department of Pediatrics, Baylor College of Medicine, Houston, TX, USA; d Development, Disease Models & Therapeutics Graduate Program, Baylor College of Medicine, Houston, TX, USA; e Jan and Dan Duncan Neurological Research Institute at Texas Children's Hospital, Houston, TX, 77030, USA

**Keywords:** Deep brain stimulation, Cerebellar nuclei, Dystonia, Sleep, Circadian rhythms

## Abstract

Dystonia arises with cerebellar dysfunction, which plays a key role in the emergence of multiple pathophysiological deficits that range from abnormal movements and postures to disrupted sleep. Current therapeutic interventions typically do not simultaneously address both the motor and non-motor symptoms of dystonia, underscoring the necessity for a multi-functional therapeutic strategy. Deep brain stimulation (DBS) is effectively used to reduce motor symptoms in dystonia, with existing parallel evidence arguing for its potential to correct sleep disturbances. However, the simultaneous efficacy of DBS for improving sleep and motor dysfunction, specifically by targeting the cerebellum, remains underexplored. Here, we test the effect of cerebellar DBS in two genetic mouse models with dystonia that exhibit sleep defects—*Ptf1a*^*Cre*^*;Vglut2*^*fx/fx*^ and *Pdx1*^*Cre*^*;Vglut2*^*fx/fx*^—which have overlapping cerebellar circuit miswiring defects but differing severity in motor phenotypes. By targeting DBS to the fiber tracts located between the cerebellar fastigial and the interposed nuclei (FN ​+ ​INT-DBS), we modulated sleep dysfunction by enhancing sleep quality and timing. This DBS paradigm improved wakefulness and rapid eye movement sleep in both mutants. Additionally, the latency to reach REM sleep, a deficit observed in human dystonia patients, was reduced in both models. Cerebellar DBS also induced alterations in the electrocorticogram (ECoG) patterns that define sleep states. As expected, DBS reduced the severe dystonic twisting motor symptoms that are observed in the *Ptf1a*^*Cre*^*;Vglut2*^*fx/fx*^ mice. These findings highlight the potential for using cerebellar DBS to simultaneously improve sleep and reduce motor dysfunction in dystonia and uncover its potential as a dual-effect *in vivo* therapeutic strategy.

## Introduction

Dystonia is comprised of an array of motor disorders and symptoms that are characterized by involuntary over-contractions and/or co-contractions of the muscles. The resulting changes culminate in abnormal postures and movements, which ultimately impose a considerable burden on the quality of life of affected individuals [1,2]. The pathophysiological underpinnings of dystonia are complex and remain only partially resolved. Contributing to this complexity is the multifaceted etiology of dystonia, ranging from hereditary factors to cerebral trauma; with a significant number of isolated dystonia cases being deemed idiopathic [2,3]. Central to our current understanding of dystonia's pathology is the “dystonia network”, an intricate network of interconnected brain regions. Functional defects in this network are implicated in dystonia in both clinical cases and animal models [4,5]. Notably, within this network, the cerebellum has emerged as a pivotal node in the manifestation of dystonic behavior. Recent research in mouse models has started to elucidate the mechanisms for how the symptoms of dystonia might initiate in response to cerebellar dysfunction [[6], [7], [8]], with corroborating evidence observed in human patients [4,9], especially within the context of the cerebello-thalamo-cortical circuit [10]. Interestingly, in addition to its prominent role in dystonia, cerebellar dysfunction has also been linked to specific impairments in sleep timing and quality [3,11,12]. Indeed, individuals with dystonia frequently experience sleep disturbances, including insomnia (indicative of a difficulty in falling asleep), parasomnias (indicative of a difficulty in staying asleep), and notably, pronounced deficits in the duration and quality of rapid eye movement (REM) sleep [[13], [14], [15]]. Analogous observations are reported in mouse models of dystonia, in which mutant mice exhibit an increase in awake time (driven by longer wake bouts), diminished REM duration (driven by fewer REM bouts), and increased latency to initiate REM sleep [16]. These data underscore the multifaceted role of the cerebellum in dystonia and suggest that it may have a broader neurological impact that extends beyond motor control to include non-motor behaviors such as the regulation of sleep.

Dystonia-associated sleep dysregulation is known to contribute to a myriad of comorbid disorders [17,18] and it can also directly worsen the motor symptoms inherent to the disease [14,19]. Consequently, a prevailing challenge in dystonia therapeutics involves the simultaneous management of both motor and non-motor (sleep-related) symptoms. Deep brain stimulation (DBS) has been widely accepted as a viable treatment option to mitigate the motor symptoms in dystonia [[20], [21], [22], [23]]. Alongside its role in addressing motor dysfunction, DBS also holds promise for the improvement of sleep disturbances, demonstrated by its potential to sustain the sleep state in which it is applied [[24], [25], [26], [27]]. While the precise mechanism(s) through which DBS improves neural dysfunction remains unclear, the leading hypotheses suggest that DBS could be enhancing, silencing, or disrupting aberrant cellular signaling [28]. Although the conventional targets for DBS in human dystonia patients remain mainly limited to basal ganglia and thalamic structures, cerebellar DBS is increasingly being explored. This is not only due to the role of the cerebellum in motor disease pathology, but also its apparent efficacy as a therapeutic target in motor diseases such as dystonia and ataxia, in which the globus pallidus and sub-thalamic targets have provided limited therapeutic benefit for some patients [29]. Indeed, we previously showed that DBS targeted to the cerebellar interposed nucleus significantly reduces the severe motor symptoms in dystonic mice and promising results have been reported in hemidystonia patients who received cerebellar dentate nucleus DBS [7,20,30].

Recent studies, including our own, propose a bidirectional role for the cerebellum in dystonia, influencing both motor and non-motor (sleep) impairments [11,16]. Given this dual function, the cerebellum emerges as an appealing target for DBS intervention to address both motor and sleep dysfunctions in dystonia. Therefore, here, we explore the potential of cerebellar nuclei DBS as a multifunctional therapeutic strategy, which could be used to manage both motor and sleep dysfunctions in dystonia. Toward this, we examined the *Ptf1a*^*Cre*^*;Vglut2*^*fx/fx*^ and *Pdx1*^*Cre*^*;Vglut2*^*fx/fx*^ mouse models of dystonia. We focused on the impact of modulating the cerebellar circuit with DBS on sleep and motor disturbances, while accounting for the differences in the severity of motor symptoms and cerebellar circuitry changes in each model. Each model showcases a specific severity in its overall motor phenotypes (severe dystonia in *Ptf1a*^*Cre*^*;Vglut2*^*fx/fx*^ mice including persistent twisting postures and tremor, with no such overt and unprovoked motor symptoms in *Pdx1*^*Cre*^*;Vglut2*^*fx/fx*^ mice). The differences in dystonic phenotypes are driven primarily by differential expression patterns of *Ptf1a* and *Pdx1* in the cerebellar circuit [7,8], leading to shared yet different degrees of silencing the olivocerebellar input to Purkinje cells, which drives dystonic motor behaviors and is a circuit implicated in human dystonia [31]. Together, these models provide a unique platform for comparative analysis and for tracking how cerebellar DBS affects motor dysfunction and sleep; their patterns, timing, and quality of sleep were recently reported [16].

In this work, we test whether cerebellar fastigial and interposed nuclei DBS (FN ​+ ​INT-DBS, which targets the tracts between the two nuclei) can reduce both motor and sleep deficits in addition to motor benefits observed with DBS in the interposed nuclei only (INT-DBS). In the absence of the stimulation, *Ptf1a*^*Cre*^*;Vglut2*^*fx/fx*^ and *Pdx1*^*Cre*^*;Vglut2*^*fx/fx*^ mice display increased awake time, increased non-REM (NREM) time, and decreased REM time. While INT-DBS successfully reduced the increased electromyography (EMG) power in the *Ptf1a*^*Cre*^*;Vglut2*^*fx/fx*^ mutant mice with severe dystonic motor symptoms, it failed to correct the sleep changes observed in both groups of mice. This suggests that restoring motor function alone does not correct sleep changes and highlights the need for a better dual-function therapeutic target. Interestingly, FN ​+ ​INT-DBS restored awake and REM sleep time. FN ​+ ​INT-DBS also normalized REM latency in both groups of mutant mice, which was elevated prior to stimulation, similar to human patients with dystonia [13]. Spectral analysis of ECoG signals also showed that the FN ​+ ​INT-DBS induced alterations in spectral power in frequency bands that are relevant for defining sleep states, indicative of changes in sleep physiology. Additionally, in the *Ptf1a*^*Cre*^*;Vglut2*^*fx/fx*^ mice, FN ​+ ​INT-DBS reduced their elevated EMG power during all stages of sleep, indicating that motor function was normalized during sleep. This work suggests that the cerebellum, and in particular its specific neural pathways, could contain the ideal circuit locus to serve as a dual therapeutic target for restoring movement and sleep, and that the application of cerebellar FN ​+ ​INT-DBS may be a viable option to fill an existing gap in treating multiple behavioral deficits in dystonia.

## Methods

### Animals

All mice used in this study were housed in a Level 3, AALAS-certified facility. All experiments and studies that involved mice were reviewed and approved by the Institutional Animal Care and Use Committee of Baylor College of Medicine (BCM AN-5996). Dr. Chris Wright (Vanderbilt University School of Medicine) kindly provided the *Ptf1a*^*Cre*^ mice and Dr. Qingchun Tong (The University of Texas Health Science Center at Houston) provided the *Pdx1*^*Cre*^ mice [32]. We purchased the *Vglut2*^*floxed*^ (*Vglut2*^*fx*^, #012898) mice from The Jackson Laboratory (Bar Harbor, ME, USA) and then maintained them in our colony using a standard breeding scheme. The conditional knock-out mice that resulted in dystonia were generated by crossing *Ptf1a*^*Cre*^*;Vglut2*^*fx/+*^ heterozygote mice or *Pdx1*^*Cre*^*;Vglut2*^*fx/+*^ heterozygote mice with homozygote *Vglut2*^*fx/fx*^ mice. The *Pdx1*^*Cre*^*;Vglut2*^*fx/fx*^ and *Ptf1a*^*Cre*^*;Vglut2*^*fx/fx*^ mice were considered experimental animals. A full description of the genotyping details (e.g., primer sequences and the use of a standard polymerase chain reaction) and phenotype for the *Ptf1a*^*Cre*^*;Vglut2*^*fx/fx*^ mouse was provided in White and Sillitoe, 2017 [7]. A full description of the genotype and the initial observations of the phenotype of the *Pdx1*^*Cre*^*;Vglut2*^*fx/fx*^ mutant mouse was described in Lackey, 2022 [8]. All littermates lacking Cre upon genotyping were considered control mice. Ear punches were collected before weaning and used for genotyping and identification of the different alleles. For all experiments, we bred mice using standard timed pregnancies, noon on the day a vaginal plug was detected was considered embryonic day (E)0.5 and postnatal day (P)0 was defined as the day of birth. Adult mice (between 4 and 6 months of age) of both sexes were used, and the data were combined in all experiments.

### Surgical procedures

Prior to surgery, mice were given preemptive analgesics (buprenorphine, 1 ​mg/kg subcutaneous (SC), and meloxicam, 5 ​mg/kg SC) with continued application as part of the standard 3 day post-operative procedure. Mice were anesthetized with isoflurane and placed into a stereotaxic device, which continued to deliver isoflurane throughout the surgery. Each mouse was implanted with a prefabricated ECoG/EMG headmount (Pinnacle Technology, Lawrence KS, #8201) with 0.10” EEG screws to secure headmounts to the skull (Pinnacle Technology, Lawrence KS, #8209). A midline incision was made, and the skull was exposed. The headmount was affixed to the skull using cyanoacrylate glue to hold in place while pilot holes for screws were made and screws were inserted. Screws were placed bilaterally over the parietal cortex and frontal cortex. A small amount of silver epoxy (Pinnacle Technology, Lawrence KS, #8226) was applied to the screw-headmount connection. Platinum-iridium EMG wires on the prefabricated headmount were placed under the skin of the neck, resting directly on the trapezius muscles.

During the same surgery to implant ECoG/EMG headmounts, two small craniotomies were performed bilaterally through which two custom 50 ​mm twisted bipolar Tungsten DBS electrodes were lowered just into the region of interest (PlasticsOne, Roanoke, VA, USA; #8IMS303T3B01). The following coordinates were used: −6.48 ​mm AP, ±1.25 ​mm ​ML, −2.25 ​mm DV from surface of the brain for the region between the fastigial and interposed nuclei (FN ​+ ​INT) and −6.4 ​mm AP, ±1.3 ​mm ​ML, −2.0 ​mm DV from surface of the brain for the interposed nuclei (INT). Nissl staining was used to confirm the final probe positions *post-hoc*, with an acceptable range of ±0.1–0.15 ​mm AP/ML/DV from the surgery coordinates.

The headmount and DBS electrodes were permanently affixed to the skull using ‘Cold-Cure’ dental cement (A-M systems, #525000 and #526000). Mice were allowed to recover for 3–4 days before being fitted with a preamplifier (Pinnacle Technology, Lawrence KS, #8202) and tethered to the recording device (Pinnacle Technology, Lawrence KS, #8204 and #8206-HR) and the deep brain stimulation device (Multi Channel Systems, #STG4002).

### Tissue preparation and histology

For perfusion fixation, animals were deeply anesthetized with 2, 2, 2-tribromoethanol (Avertin), and then perfused through the heart with 0.1 ​m PBS (pH 7.2), followed by 4 ​% paraformaldehyde (PFA) diluted in PBS. The brains from the perfused mice were postfixed for 24–48 ​h in 4 ​% PFA and then cryoprotected stepwise in PBS-buffered sucrose solutions (15 ​% and 30 ​% each time until the brain sunk). Serial 40-μm-thick coronal tissue sections were cut on a cryostat, and then each tissue section collected in PBS and processed free floating.

Nissl staining was performed by first mounting the tissue sections on slides and allowing them to dry on the slides overnight. Mounted sections were immersed in 100 ​% xylene two times for 5 ​min and then put through a rehydration series, which consisted of 3 immersions in 100 ​% ethanol followed by 95 ​% ethanol, 70 ​% ethanol, 50 ​% ethanol and then tap water, for 2 ​min per step. The tissue sections were then immersed in cresyl violet solution for ∼30 ​s or until the stain was sufficiently dark. The tissue sections were then dehydrated using the reversed order of the rehydration series (described above) followed by xylene, with 30 s per step. Cytoseal XYL mounting media (Thermo Scientific, Waltham, MA, USA, #22-050-262) and a coverslip was then immediately placed on the slides.

### ECoG/EMG sleep recording and DBS protocol

**Fig. 1:**
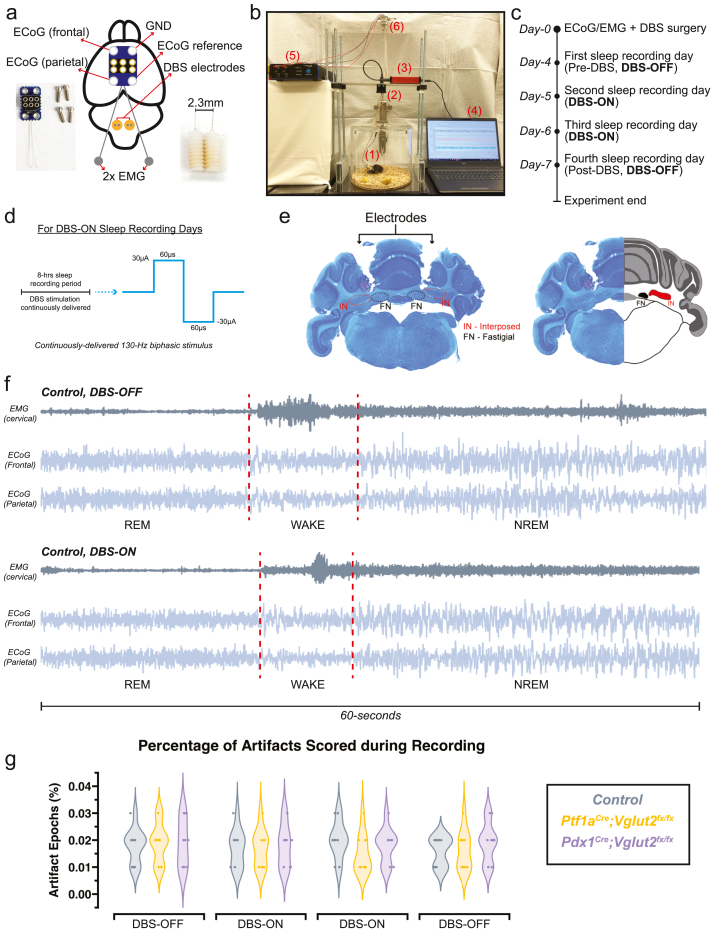
**Simultaneous recordings of ECoG and EMG during cerebellar nuclei DBS** **(a)** A schematic illustration of a mouse brain showing the placement of the ECoG/EMG headmount and DBS electrodes. An image of the ECoG/EMG headmount and screws is shown in the bottom left. An image of the DBS electrodes is shown in the bottom right. **(b)** Video still from a sample sleep recording showing the experimental setup while a mouse is being recorded and stimulated. The ECoG/EMG preamplifier tether is shown in (1). The 360^o^ commutator is shown in (2). The data acquisition system is shown in (3). All ECoG/EMG data is recorded with a PC computer and stored for analysis as shown in (4). The DBS stimulator box is shown in (5). The 360^o^ commutator for the DBS tether is shown in (6). **(c)** A schematic illustration of the experimental timeline for recording sleep. **(d)** A schematic illustration of the DBS parameters. **(e)** A Nissl stain showing the bilateral placement of DBS electrodes between the FN and INT, and an illustration showing the relative anatomical locations of these structures in the brain. **(f)** Raw ECoG/EMG recordings from a control mouse, showing normal waveforms in the presence and absence of DBS during recording. **(g)** Quantification of the percentage of artifacts scored during sleep recordings to capture the degree of signal corruption in the recordings and assess recording data quality. Points on **(g)** represent individual mice (n ​= ​9 per group). Source data and specific *p*-values for **g** are shown in Fig. 1 – source data 1.

Mice were recorded in light and temperature-controlled rooms at the same time of day for every mouse and for each day. Sleep was recorded continuously for 8 ​h during the light phase (when mice naturally sleep) for four consecutive days, so that sleep quality could be assessed pre-DBS, during DBS, and post-DBS (Fig. 1b and c). The first hour of recording was considered the acclimation period and was therefore excluded from final analysis. Food and water were available *ad libitum* throughout the recording days. Mice were tethered to both the sleep recording hardware and the DBS stimulator for each day of recording. The DBS stimulator was turned on for every day of recording, but stimulation was delivered only on days labeled “DBS ON” (second and third sleep recording days) and omitted for “DBS OFF” days (first and fourth sleep recording days). On the days when the DBS was delivered, the mice were stimulated continuously for the entire duration of the 8-h recording period, with a 130 ​Hz biphasic stimulus (30 ​μA, 60 μs duration) similar to previous work [7] (Fig. 1d).

### Sleep scoring and analysis of sleep data

Sleep stages and artifacts were automatically scored offline with ‘Sleep Phase Identification with Neural networks for Domain-invariant LEearning’ (SPINDLE) [33], as previously described [16,34]. In brief, raw files were binned in 10-s intervals and pre-processed in MATLAB (MathWorks) using the free toolkit EEGLAB [35] to obtain power spectra (Welch's method, with a 50 ​% overlapping window) of both ECoG and EMG signals. The average of values measured from the frontal and parietal cortexes was used to calculate the following ECoG band power values: delta (0.5–4 ​Hz) frequency band (a feature of NREM sleep); theta (5–8 ​Hz) frequency band (a feature of REM sleep); and alpha (8–13 ​Hz) frequency band (a feature of wake). SPINDLE then classifies the bin as ‘wake’ if either the alpha power/(theta power/delta power) value exceeds one standard deviation above the average for the animal or EMG root mean square (RMS) exceeds 0.4 standard deviations above the mean. The bin is categorized as ‘sleep’ when neither threshold is met. Sleep bins are further classified as ‘REM sleep’ if theta-to-delta power ratio exceeds one standard deviation above the average for the animal and as ‘NREM sleep’ for all other sleep bins. In addition, SPINDLE identifies ‘artifacts’ by operating a convolutional neural network to learn discriminative and translation-invariant features of time-frequency representations of ECoG/EMG and a hidden Markov model to predict vigilance state transition dynamics that are physiologically infeasible. The percentage of signal artifacts signifies the degree of signal corruption in the recordings to help assess recording data quality. Scored files were downloaded from SPINDLE as a.csv and statistical analysis was performed in R v4.1.2.

### Data analysis and statistics

Data are presented as mean ​± ​SEM and analyzed as a mixed ANOVA followed by post-hoc pairwise comparisons with Tukey's method for multiple comparisons correction. *p* ​< ​0.05 was considered as statistically significant. All statistical analyses were performed using R v4.1.2.

## Results

### Targeting the cerebellum with DBS does not impair the acquisition of ECoG/EMG recordings, nor the ability to classify arousal states

Previously, in a series of independent studies, we (1) employed ECoG/EMG recordings to evaluate sleep quality and (2) used cerebellar DBS to mitigate motor dysfunction in mouse models [7,16,20,30]. However, the unified application of both techniques (ECoG/EMG recordings and cerebellar DBS) requires validation to ensure that they would not adversely interfere with our ability to measure the functional outcomes *in vivo*. We therefore implanted *Ptf1a*^*Cre*^*;Vglut2*^*fx/fx*^ and *Pdx1*^*Cre*^*;Vglut2*^*fx/fx*^ mice with prefabricated ECoG/EMG headmounts, and with twisted bipolar Tungsten DBS electrodes. The ECoG/EMG headmount was oriented such that the recording electrodes were situated above the frontal and parietal cortices, and the DBS electrode was oriented above the cerebellum (Fig. 1a). DBS electrodes were targeted bilaterally to terminate between the interposed and fastigial cerebellar nuclei, due to their roles in movement-regulation [36] and connections to sleep [[37], [38], [39]], respectively. The electrodes terminated ∼2.5 ​mm from the surface of the brain, within the cerebellar white matter just above the nuclei core. (Fig. 1e). Sleep quality was assessed from the pre-DBS sleep recording day, two DBS-ON days, followed by the final DBS-OFF day (Fig. 1b–d). Raw ECoG/EMG waveforms showed that the overall DBS paradigm and targeting did not affect the quality of the recording, nor did it affect the ability to classify the stages of wake, REM, or NREM sleep (Fig. 1f). To confirm the integrity of the recorded data, we quantified the total number of artifacts observed across all mice in each group. Artifacts were automatically noted during offline sleep scoring (see Methods). Our analysis did not reveal significant differences in the proportion of artifacts scored across recording days, ensuring the consistency and reliability of our experimental setup and data collection (Fig. 1g).

### FN ​+ ​INT-DBS decreases total wake time and increases total REM and NREM time in *Ptf1a*^*Cre*^*;Vglut2*^*fx/fx*^ and *Pdx1*^*Cre*^*;Vglut2*^*fx/fx*^ mice

**Fig. 2:**
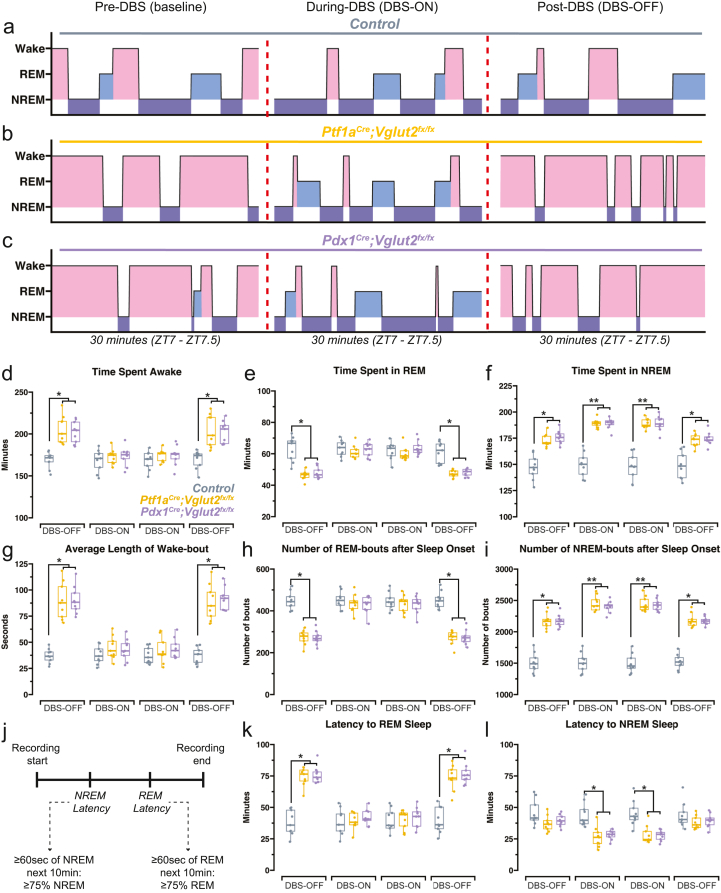
**FN ​+ ​INT-DBS impacts the same measures of sleep quality in both the *Ptf1a*^*Cre*^*;Vglut2*^*fx/fx*^ and the *Pdx1*^*Cre*^*;Vglut2*^*fx/fx*^ mutant mice** **(a-c)** Hypnograms taken from three different recording days, for a single representative control mouse **(a)**, *Ptf1a*^*Cre*^*;Vglut2*^*fx/fx*^ mouse **(b)**, and *Pdx1*^*Cre*^*;Vglut2*^*fx/fx*^ mouse **(c)** for the same 30-min period, ZT7-7.5, where ZT0 ​= ​lights ON, ZT1 ​= ​1 ​h after lights ON, etc. Periods of wake are highlighted in red, periods of REM are highlighted in light blue, periods of NREM are highlighted in dark purple. Dotted red lines separate different recording days, with different DBS status. **(b)** Same as **(a)** but for a *Ptf1a*^*Cre*^*;Vglut2*^*fx/fx*^ mouse. **(c)** Same as **(a)** but for a *Pdx1*^*Cre*^*;Vglut2*^*fx/fx*^ mouse. **(d)** Quantification of total time spent awake. **(d**–**l)** FN ​+ ​INT-DBS rectified the increase in awake time **(d)** and bouts **(g)** and also normalized the decrease in REM sleep time **(e)** and bouts **(h)** that were observed in the mutant mice. However, its benefits did not persist on the final sleep recording day (without stimulation, DBS-OFF). Legend showing control mice in gray, *Ptf1a*^*Cre*^*;Vglut2*^*fx/fx*^ mice in gold, *Pdx1*^*Cre*^*;Vglut2*^*fx/fx*^ mice in purple applies to **d-i, k and l**. In contrast, FN ​+ ​INT-DBS had no significant effect on the elevated NREM time **(f)** and bouts **(i)**. Upon examining NREM and REM latency as described in Hunsley & Palmiter, 2004 [65] **(j)**, FN ​+ ​INT-DBS normalized the increase in REM latency **(k)** and shortened NREM latency **(l)**. Points on **(d-i,k and l)** represent individual mice (n ​= ​9 per group). Source data and specific *p*-values for **d-i, kandl** are shown in Fig. 2 – source data 1. ∗*p* ​≤ ​0.05, ∗∗*p* ​≤ ​0.01.

The relationship between sleep and motor function is crucial in dystonia. Observations have shown that after a good night's sleep, motor symptoms become more manageable. This improvement is especially notable in the early morning after awakening [1,13]. However, existing therapies fail to improve sleep in patients [14]. Consequently, a primary objective of our study was to investigate whether FN ​+ ​INT-DBS might improve overall sleep quality in *Ptf1a*^*Cre*^*;Vglut2*^*fx/fx*^ and *Pdx1*^*Cre*^*;Vglut2*^*fx/fx*^ mice. At baseline (Pre-DBS), representative hypnograms indicate that both *Ptf1a*^*Cre*^*;Vglut2*^*fx/fx*^ and *Pdx1*^*Cre*^*;Vglut2*^*fx/fx*^ mice display overall greater time awake and less time asleep relative to controls, similar to our previous findings [16]. Remarkably, during DBS, the proportions of wakefulness and sleep time began to converge across all mouse groups, demonstrating a rectification of sleep dysfunction. However, post-DBS ECoG recordings, when stimulation was withheld (fourth sleep recording day), indicated that the sleep dysfunctions re-emerged in both *Ptf1a*^*Cre*^*;Vglut2*^*fx/fx*^ and *Pdx1*^*Cre*^*;Vglut2*^*fx/fx*^ mice (Fig. 2a–c). We quantified the time spent in each state and found that indeed, DBS rectified the sleep deficits in the mutant mice. Both *Ptf1a*^*Cre*^*;Vglut2*^*fx/fx*^ and *Pdx1*^*Cre*^*;Vglut2*^*fx/fx*^ mice exhibited greater total sleep time, in response to cerebellar nuclei DBS. Notably, these changes demonstrated high temporal fidelity to DBS, as sleep-wake patterns reverted to baseline levels in the absence of stimulation (Fig. 2d–f). We also found that DBS decreased the average length of wake bouts, increased the number of REM bouts, and increased the number of NREM bouts in *Ptf1a*^*Cre*^*;Vglut2*^*fx/fx*^ and *Pdx1*^*Cre*^*;Vglut2*^*fx/fx*^ mice (Fig. 2g–i). Furthermore, we calculated the latency to reach both REM and NREM sleep (Fig. 2j). Our findings indicated that both groups of mutant mice, *Ptf1a*^*Cre*^*;Vglut2*^*fx/fx*^ and *Pdx1*^*Cre*^*;Vglut2*^*fx/fx*^, experienced reduced latencies to REM and NREM sleep during DBS (Fig. 2k and l). These findings indicate that FN ​+ ​INT-DBS effectively improves sleep timing and quality in both *Ptf1a*^*Cre*^*;Vglut2*^*fx/fx*^ and *Pdx1*^*Cre*^*;Vglut2*^*fx/fx*^ mice, with improvements closely tied to the DBS timing. Echoing our previous research [16], these results point to cerebellar dysfunction, rather than motor impairment alone, as the likely origin of sleep deficits, given that both mutant mouse types, regardless of their motor condition, showed comparable sleep improvements during DBS.

### Delta, beta, and gamma spectral frequency oscillations are modulated by FN ​+ ​INT-DBS

**Fig. 3:**
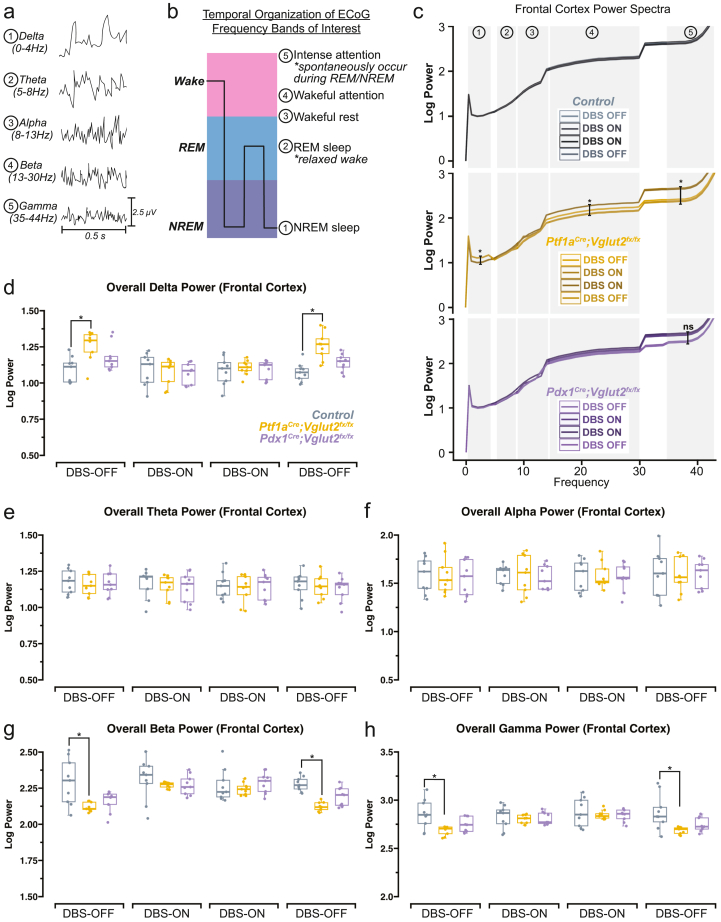
**FN ​+ ​INT-DBS normalizes differences in spectral frequency oscillations in the *Ptf1a*^*Cre*^*;Vglut2*^*fx/fx*^ and the *Pdx1*^*Cre*^*;Vglut2*^*fx/fx*^ mutant mice compared to control mice** **(a)** 1-s samples of raw ECoG waveforms for frequency bands of interest, from a control mouse. **(b)** Schematic illustration showing the relative arousal state in which different spectral frequency oscillations occur. **(c)** Upon analysis of average power spectra of the frontal cortex across the entire recording period for each mouse, FN ​+ ​INT-DBS normalized the increase in delta power **(d)** and decrease in beta **(g)** and gamma **(h)** power in the *Ptf1a*^*Cre*^*;Vglut2*^*fx/fx*^ mice. No other significant effects were observed in the remaining bands (theta **(e)** and alpha **(f)**), in other mice, nor without stimulation (DBS-OFF). Points on **(d**–**h)** represent individual mice (n ​= ​9 per group). Source data and specific *p*-values for **d-h** are shown in Fig. 3 – source data 1. ns ​= ​not significant (*p* ​> ​0.05), ∗*p* ​≤ ​0.05.

While cerebellar DBS has been established as an effective intervention for improving sleep in mouse models with dystonia, the underlying neurophysiological mechanisms remain an area of exploration. In particular, the modulation of spectral frequency oscillations during sleep provides a crucial window into understanding the intricacies of this therapeutic impact. Subsequently then, we sought to determine whether FN ​+ ​INT-DBS specifically influences these spectral frequency oscillations, which would shed light on its role in enhancing sleep quality. Arousal states are partially characterized by spectral frequency oscillations spanning a range from 0.5 to >100 ​Hz (Fig. 3a). Thus, shifts between sleep stages can be marked by fluctuations in delta (0.5–4 ​Hz in mice), theta (5–8 ​Hz in mice), or alpha (8–13 ​Hz in mice) power, suggesting either an enhancement or deterioration in sleep quality [40,41]. Beta (13–30 ​Hz in mice) and gamma (35–44 ​Hz in mice) frequency bands can also act as indicators of sleep homeostasis, as they too tend to occur during specific arousal states (Fig. 3b). We hypothesized that the existing differences in spectral power across the frequency bands of interest could be modulated alongside the patterns of sleep disruption during DBS. Upon analysis, we noted that indeed the baseline (pre-DBS) increase in frontal delta power in *Ptf1a*^*Cre*^*;Vglut2*^*fx/fx*^ mice was significantly reduced during DBS (Fig. 3c ​and d). Although the delta power in *Pdx1*^*Cre*^*;Vglut2*^*fx/fx*^ mice was elevated during baseline, but not to the point of a significant difference, DBS did reduce delta power in these mice as well, matching the levels observed in *Ptf1a*^*Cre*^*;Vglut2*^*fx/fx*^ mice and controls. Theta and alpha power remained unaltered for all mice across all conditions (Fig. 3e and f). We also observed a reduction in pre-DBS beta and gamma power in the *Ptf1a*^*Cre*^*;Vglut2*^*fx/fx*^ mice, which was normalized during DBS, as were the non-significant reductions in the *Pdx1*^*Cre*^*;Vglut2*^*fx/fx*^ mice (Fig. 3g and h). These data imply that the regulatory impacts of FN ​+ ​INT-DBS on neural circuitry extend beyond the cerebellum in this particular paradigm, evidenced by the modulation of the baseline changes in spectral frequency oscillations recorded in the *Ptf1a*^*Cre*^*;Vglut2*^*fx/fx*^ and *Pdx1*^*Cre*^*;Vglut2*^*fx/fx*^ mice.

### The increased EMG power observed in *Ptf1a*^*Cre*^*;Vglut2*^*fx/fx*^ mice is significantly reduced during FN ​+ ​INT-DBS, in all stages of sleep

**Fig. 4:**
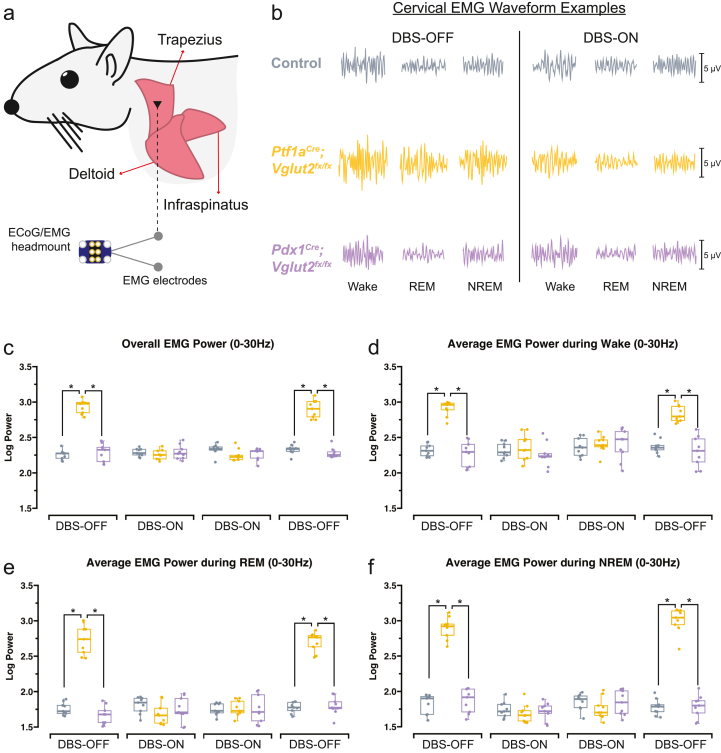
**FN ​+ ​INT-DBS reverses the abnormally increased EMG power of all sleep states observed in the *Ptf1a*^*Cre*^*;Vglut2*^*fx/fx*^ mutant mice** **(a)** Schematic illustration of a mouse showing the musculature and relative placement of the EMG electrodes. **(b)** Examples of raw EMG waveforms of trapezius activity for wake, REM and NREM sleep demonstrate high-amplitude EMG waveforms in the *Ptf1a*^*Cre*^*;Vglut2*^*fx/fx*^ mice, which shifted to resemble those in control and *Pdx1*^*Cre*^*;Vglut2*^*fx/fx*^ mice with FN ​+ ​INT-DBS. **(c)** Overall EMG activity (in the 0–30 ​Hz frequency band) was significantly reduced in the *Ptf1a*^*Cre*^*;Vglut2*^*fx/fx*^ mice during FN ​+ ​INT-DBS but returned to elevated levels in the absence of stimulation (DBS-OFF). FN ​+ ​INT-DBS reduced overall EMG power in *Ptf1a*^*Cre*^*;Vglut2*^*fx/fx*^ mice across all sleep stages: wake **(d),** REM sleep **(e),** and NREM sleep **(f).** Points on **(c**–**f)** represent individual mice (n ​= ​9 per group). Source data and specific *p*-values for **c-f** are shown in Fig. 4 – source data 1. ∗*p* ​≤ ​0.05.

The persistence of dystonia's motor dysfunction during sleep is debated, with conflicting human studies suggesting both the resolution and possible continuation of these deficits impacting sleep quality [1,13,14]. Our previous study noted elevated trapezius EMG power in *Ptf1a*^*Cre*^*;Vglut2*^*fx/fx*^ mice during both REM and NREM sleep, indicating ongoing motor symptoms [16]. We anticipated that cerebellar DBS, known for mitigating dystonic motor symptoms, would normalize these elevated EMG values. We recorded EMG signals using platinum-iridium wires that were surgically inserted into the trapezius muscles (Fig. 4a), which we used to collect data continuously throughout the sleep recording period. By analyzing the power of the recorded raw trapezius muscle activity waveforms, specific to sleep stages before and during DBS, we showed that the stimulation normalized EMG activity. Specifically, the typically high-amplitude EMG waveforms in the *Ptf1a*^*Cre*^*;Vglut2*^*fx/fx*^ mice during DBS shifted to more closely resemble those in control and *Pdx1*^*Cre*^*;Vglut2*^*fx/fx*^ mice, both of which are devoid of motor dysfunction (Fig. 4b). We calculated overall EMG power in the 0–30 ​Hz frequency band, previously used to diagnose dystonia in human patients [42], and found that overall EMG activity was significantly reduced in the *Ptf1a*^*Cre*^*;Vglut2*^*fx/fx*^ mice during DBS. Additionally, overall EMG power returned to baseline (elevated) levels in the absence of stimulation (Fig. 4c). We further calculated EMG power only during periods of wake, REM, or NREM sleep and found that EMG power was similarly reduced in all states, in *Ptf1a*^*Cre*^*;Vglut2*^*fx/fx*^ mice, with high temporal fidelity to DBS (Fig. 4d–f). These findings confirm our previous work showing that, in the absence of DBS, dystonic muscle activity in *Ptf1a*^*Cre*^*;Vglut2*^*fx/fx*^ mice persists in all sleep stages. Additionally, they suggest that our FN ​+ ​INT-DBS paradigm can move the elevated EMG power toward normalcy in *Ptf1a*^*Cre*^*;Vglut2*^*fx/fx*^ mice, suggesting that correlates of the dystonic motor signatures are alleviated in all sleep states.

### INT-DBS normalizes EMG and frontal cortex beta power but does not rescue the sleep changes in *Ptf1a*^*Cre*^*;Vglut2*^*fx/fx*^ and *Pdx1*^*Cre*^*;Vglut2*^*fx/fx*^ mice

**Fig. 5:**
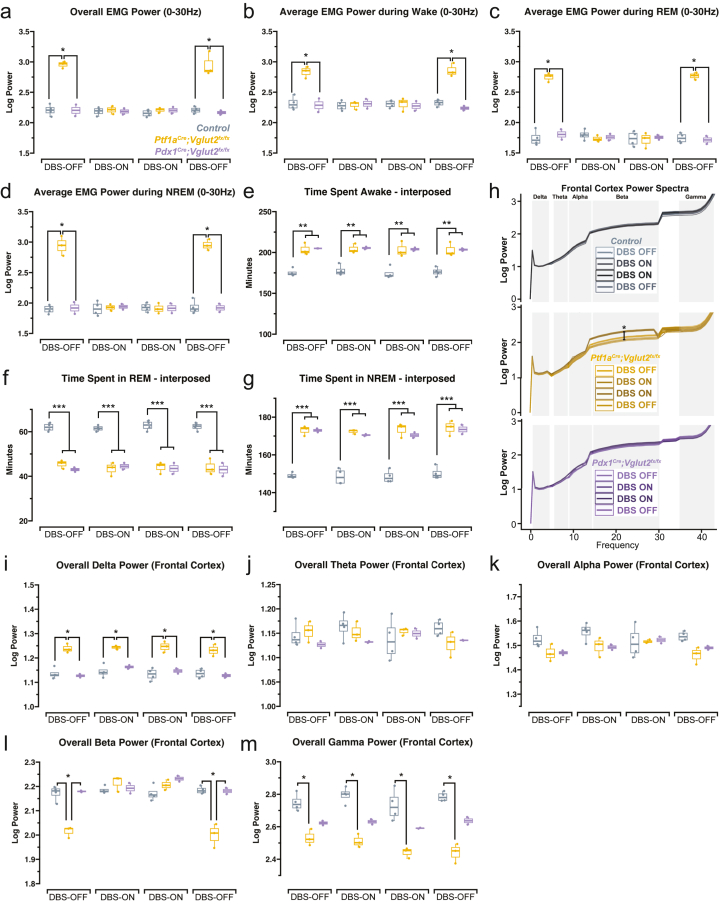
**INT-DBS does not correct the sleep changes observed in both types of mutant mice while reversing abnormally increased EMG power of all sleep states in the *Ptf1a*^*Cre*^*;Vglut2*^*fx/fx*^ mutant mice** **(a)** Overall EMG power, which was elevated in the *Ptf1a*^*Cre*^*;Vglut2*^*fx/fx*^ mice at baseline, was normalized with INT-DBS but its effect did not persist without stimulation (DBS-OFF). The reduction in overall EMG power of the *Ptf1a*^*Cre*^*;Vglut2*^*fx/fx*^ mice with INT-DBS was observed across all sleep stages: wake **(b)**, REM sleep **(c)**, and NREM sleep **(d)**. **(e)** The increase in time spent awake among the *Ptf1a*^*Cre*^*;Vglut2*^*fx/fx*^ and *Pdx1*^*Cre*^*;Vglut2*^*fx/fx*^ mice was not corrected by INT-DBS. **(f)** INT-DBS failed to bring time spent in REM sleep back to normalcy in both types of mutant mice. **(g)** INT-DBS did not reduce the elevated time spent in NREM sleep that was observed in both types of mutant mice. **(h)** Analysis of average power spectra of the frontal cortex demonstrated that INT-DBS increased beta power in the *Ptf1a*^*Cre*^*;Vglut2*^*fx/fx*^ mice and therefore modulated it to match the normal range seen in controls and *Pdx*^*Cre*^*;Vglut2*^*fx/fx*^ mice **(l)**. No other significant effects of INT-DBS were observed in the remaining bands (delta **(i)**, theta **(j)**, alpha **(k)**, and gamma **(m)**). Points on **(a-g, i-m)** represent individual mice (n ​= ​4 for control *Vglut2*^*fx/fx*^ mice, n ​= ​3 for *Ptf1a*^*Cre*^*;Vglut2*^*fx/fx*^ mice and n ​= ​2 for *Pdx1*^*Cre*^*;Vglut2*^*fx/fx*^ mice). Source data and specific *p*-values for **a-m** are shown in Fig. 5 – source data 1. ∗*p* ​≤ ​0.05, ∗∗*p* ​≤ ​0.01, ∗∗∗*p* ​≤ ​0.001.

We have previously demonstrated that sleep disruptions in mouse models of dystonia depend on cerebellar dysfunction but not motor symptoms in mice [16]. Here, we sought to test whether a reduction in the elevated EMG power that we observed in the dystonic mice could be achieved with INT-DBS to further resolve if this stimulation paradigm impacts the sleep deficits that are associated with dystonia. In alignment with our previous findings [7], INT-DBS decreased the overall elevated EMG activity observed in *Ptf1a*^*Cre*^*;Vglut2*^*fx/fx*^ mice during stimulation (Fig. 5a–d). However, in contrast to FN ​+ ​INT-DBS, the INT-DBS failed to bring abnormal sleep changes observed in both groups of mutant mice back to normalcy (Fig. 5e–g). This suggests that the reduction of motor symptoms alone is insufficient for restoring sleep changes back to normalcy. Moreover, spectral power analysis of the frontal cortex revealed an increase of beta power in *Ptf1a*^*Cre*^*;Vglut2*^*fx/fx*^ mice to match controls during INT-DBS, but no other changes were observed among delta, theta, alpha, and gamma bands (Fig. 5h-m). Unlike the changes we observed in the frontal cortex oscillations with FN ​+ ​INT-DBS, delta and gamma powers in the *Ptf1a*^*Cre*^*;Vglut2*^*fx/fx*^ mice remained significantly different from controls and *Pdx*^*Cre*^*;Vglut2*^*fx/fx*^ mice during INT-DBS (Fig. 5i and m). These data suggest that restoring normal sleep in dystonia requires improved communication of sleep-related activity between the cerebellum and frontal cortex.

## Discussion

In this study, we examined both the motor and sleep dysfunctions of dystonia and the potential for therapeutic cerebellar DBS to improve sleep quality in mice. We dissected the role of the cerebellum, specifically tracts between the FN and INT or the INT alone, as targets for DBS therapy in two mouse models of dystonia, *Ptf1a*^*Cre*^*;Vglut2*^*fx/fx*^ and *Pdx1*^*Cre*^*;Vglut2*^*fx/fx*^, which have similar cerebellar deficits but differing motor phenotypes. While both FN ​+ ​INT-DBS and INT-DBS reduced the elevated EMG power in *Ptf1a*^*Cre*^*;Vglut2*^*fx/fx*^ mice, INT-DBS did not rectify the sleep changes observed in both mouse models of dystonia. In contrast, during FN ​+ ​INT-DBS, we observed a reduction in wakefulness and an increase in REM sleep in both mutant groups, indicating an overall reversal of these specific sleep deficits that are associated with dystonia. Moreover, we identified a notable decrease in the latency to REM and NREM sleep, addressing a prominent sleep dysfunction commonly reported in human dystonia patients [13,14]. This work also highlights how the ECoG patterns that define sleep states are modulated by FN ​+ ​INT-DBS, providing additional insight into the multifunctional therapeutic benefits of modulating cerebellar function to improve both sleep regulation and motor function.

While typical DBS therapies for motor disorders such as dystonia often target various subcortical structures including the basal ganglia and thalamus, recent evidence has suggested that cerebellar DBS shows promise for dystonia, which can be unresponsive to standard DBS targeting in a subpopulation of human patients [21,29]. Indeed, INT-DBS rescues motor deficits in mouse models for dystonia, tremor and ataxia [7,20,30,43], but it failed to address the sleep deficits that are associated with dystonia (Fig. 5e–g). This may not be surprising given that traditional approaches, such as botulinum toxin injections and even traditional DBS, which improve the motor symptoms of dystonia, have typically fallen short in simultaneously addressing the non-motor (sleep) symptoms of the disorder [14]. As we sought to further develop cerebellar DBS as a multi-modal therapy to address sleep alongside motor deficits, we expanded our target to encompass the neighboring FN as well, given its role in non-motor functions, particularly sleep. Of the cerebellar nuclei responses with sleep-dependent changes in activity, the trends for increased firing may be strongest in the FN [38,44]. The FN is reported to have reciprocal projections with the hypothalamus, notably with the lateral hypothalamus, a region known to contribute extensively to regulating sleep-wake behavior [37]. Beyond the specific sleep-related connections of the FN, this nucleus also forms connections with the vestibular nuclei, reticular formation, and thalamus, which collectively project to many areas including the cerebral cortex. Such extensive connectivity underscores the cerebellum's profound influence on both motor and non-motor functions.

Interestingly, while wake and REM time was normalized by FN ​+ ​INT-DBS, NREM time remained elevated in both *Ptf1a*^*Cre*^*;Vglut2*^*fx/fx*^ and *Pdx1*^*Cre*^*;Vglut2*^*fx/fx*^ mice (Fig. 2f). Previous work suggests that DBS delivered during sleep could extend the sleep stage in which it is administered [24]. Given that our FN ​+ ​INT-DBS paradigm is continuous without specific temporal constraints, it raises the possibility that our stimulation indiscriminately increases all sleep states. However, in this case we would expect to see changes in sleep for control mice, which we did not. Another interpretation could be related to the “REM Rebound” phenomenon, where, following periods of sleep deprivation or significant stressors, both rodents and humans display extended and intensified REM sleep during the subsequent sleep period [45]. While it is uncertain whether a similar effect exists for NREM sleep, the observed increase in NREM time in our study could be attributed to a similar mechanism. Additionally, FN ​+ ​INT-DBS may enhance sleep pressure. Our findings do reveal a shortened latency to NREM sleep in both *Ptf1a*^*Cre*^*;Vglut2*^*fx/fx*^ and *Pdx1*^*Cre*^*;Vglut2*^*fx/fx*^ mice during FN ​+ ​INT-DBS, suggesting that mice in both groups fall asleep more rapidly during stimulation (Fig. 2l). However, if stimulation universally increased sleep pressure, we would anticipate observing the same phenomenon in control mice, which is not the case. Alternatively, instead of enhancing sleep pressure, stimulation may resolve an existing impediment to sleep pressure by correcting abnormal signaling in the cerebellar circuit of *Ptf1a*^*Cre*^*;Vglut2*^*fx/fx*^ and *Pdx1*^*Cre*^*;Vglut2*^*fx/fx*^ mice. In this case, we would anticipate the changes in NREM latency evoked by FN ​+ ​INT-DBS to be specific to the mutant mice, while the controls would be unaffected. Such a mechanism would concur with the hypothesis that, at least in some conditions and in specific circuits, FN ​+ ​INT-DBS could function by causing an “informational lesion” that impacts select synapses, ultimately correcting (or compensating for) convergent sites of dysfunction [28].

Given that there are projections between the cerebellum and regions that not only regulate sleep, but specific stages of sleep (REM and NREM) [3,46,47], we sought to determine if FN ​+ ​INT-DBS improved overall time spent sleeping via specific alterations in the length and frequency of individual wake, REM, or NREM bouts. Indeed, we previously found that the sleep deficits in the *Ptf1a*^*Cre*^*;Vglut2*^*fx/fx*^ and *Pdx1*^*Cre*^*;Vglut2*^*fx/fx*^ mice were driven by an increased wake bout length, a decreased number of REM bouts, and an increased number of NREM bouts. The same deficits were recapitulated in the current cohort of mice in the absence of stimulation at baseline and post-stimulation periods. During stimulation, we found that changes were induced in these same three measures of sleep architecture. For both *Ptf1a*^*Cre*^*;Vglut2*^*fx/fx*^ and *Pdx1*^*Cre*^*;Vglut2*^*fx/fx*^ mice during DBS, the average length of wake bouts was decreased, and the frequency of both REM and NREM bouts was increased (Fig. 2g–i). The dynamics of these specific changes in sleep architecture do suggest, as we discussed above, that FN ​+ ​INT-DBS in the *Ptf1a*^*Cre*^*;Vglut2*^*fx/fx*^ and *Pdx1*^*Cre*^*;Vglut2*^*fx/fx*^ mice is either rectifying an impediment to natural sleep pressure or causing a dual REM-rebound and NREM-rebound effect, or both. Indeed, the combination of these two possible mechanisms of sleep rectification together would explain the increase of both REM and NREM frequency, even though only REM sleep is decreased in the mutant mice at baseline.

In further support of the dual sleep pressure and sleep rebound model, we also found that specific sleep latencies were normalized by FN ​+ ​INT-DBS in both *Ptf1a*^*Cre*^*;Vglut2*^*fx/fx*^ and *Pdx1*^*Cre*^*;Vglut2*^*fx/fx*^ mice. The rectification of REM latency is of particular interest, as achieving REM sleep is often a particular challenge faced by patients with dystonia [13,14]. Although the reduction in REM latency in the *Ptf1a*^*Cre*^*;Vglut2*^*fx/fx*^ mouse model—characterized by overt dystonic motor dysfunction—could be ascribed to the alleviation of motor symptoms, the parallel reduction in latency observed in the *Pdx1*^*Cre*^*;Vglut2*^*fx/fx*^ mice, which lack overt motor symptoms, implies a more fundamental role of cerebellar dysfunction, and cerebellar DBS. This points not only to the cerebellum's integral involvement in these specific sleep impairments but also to the potential of cerebellum-focused therapeutics (invasive or non-invasive) for resolving pathophysiologically different symptoms in neurological diseases.

Our ECoG spectral activity analysis presents noteworthy findings, which illuminate the fundamental contributors to the specific sleep deficits in *Ptf1a*^*Cre*^*;Vglut2*^*fx/fx*^ and *Pdx1*^*Cre*^*;Vglut2*^*fx/fx*^ mice and the mechanism of FN ​+ ​INT-DBS in restoring sleep quality. These findings demonstrate that the effects of our FN ​+ ​INT-DBS and INT-DBS are not limited to the cerebellum; they extend to and influence cortical activity. The detected decrease in delta power during FN ​+ ​INT-DBS for *Ptf1a*^*Cre*^*;Vglut2*^*fx/fx*^ mice (Fig. 3d) concurs with independent studies that show a reduction in delta power across successive NREM sleep periods [48]. Sleep deprivation, known to trigger a surge in delta power [41], is prominent when FN ​+ ​INT-DBS is absent and the mutant mice exhibit severe sleep deficits and reduced sleep time. FN ​+ ​INT-DBS reduces wakefulness and increases sleep duration and NREM bouts (Fig. 2d–f, i), which would mitigate the state of sleep deprivation along with a reduction in delta power. Our observations of enhanced beta and gamma power during stimulation add further depth to our understanding of how FN ​+ ​INT-DBS influences sleep in the *Ptf1a*^*Cre*^*;Vglut2*^*fx/fx*^ and *Pdx1*^*Cre*^*;Vglut2*^*fx/fx*^ mice. It is known that diminished beta power is linked with suboptimal sleep quality and could potentially suggest obstructive sleep apnea [49]. Even though the presence of obstructive sleep apnea in the *Ptf1a*^*Cre*^*;Vglut2*^*fx/fx*^ mice has not been determined, we interpret the increase in beta power during stimulation (Fig. 3g) as possibly indicative of a restoration of motor function, a change we know our stimulation paradigm can induce. Indeed, this interpretation is further strengthened by INT-DBS normalizing EMG and beta power in the *Ptf1a*^*Cre*^*;Vglut2*^*fx/fx*^ mice but failing to rectify delta and gamma powers nor sleep deficits (Fig. 5). Meanwhile, the rise in gamma power during FN ​+ ​INT-DBS (Fig. 3h) could stem from an overall increase in REM and NREM sleep durations. Given the known spontaneous occurrence of gamma oscillations during both REM and NREM sleep [50], the overall increase in gamma power could correspond to the lengthened REM and NREM periods during FN ​+ ​INT-DBS for the mutant mice.

Lastly, we observed that *Pdx1*^*Cre*^*;Vglut2*^*fx/fx*^ mice did not exhibit significant changes in delta, beta, or gamma power in the absence of either DBS paradigms and that power in these bands does not differ significantly from that of *Ptf1a*^*Cre*^*;Vglut2*^*fx/fx*^ mice or controls (Fig. 3d, g and h and Fig. 5i, l and m). We have previously speculated that this may be due to their ‘intermediate’ cerebellar and motor phenotype, arising from the different pattern of olivocerebellar VGLUT2 deletion relative to the *Ptf1a*^*Cre*^*;Vglut2*^*fx/fx*^ mice [16]. In this case, the subtlety in the observed spectral differences between *Ptf1a*^*Cre*^*;Vglut2*^*fx/fx*^ and *Pdx1*^*Cre*^*;Vglut2*^*fx/fx*^ mice could in fact serve as precise biomarkers of disease severity, as shifts in spectral power can vary in direction and magnitude across different diseases, even when the affected individuals all exhibit similarly disrupted sleep [41,51]. We do also recognize that spectral differences in sleep between *Ptf1a*^*Cre*^*;Vglut2*^*fx/fx*^ and *Pdx1*^*Cre*^*;Vglut2*^*fx/fx*^ mice may intertwine with alterations in the motor program, considering the known influence of movement patterns on ECoG spectral activity [52]. Nevertheless, despite their “intermediate” spectral power differences, during FN ​+ ​INT-DBS, the *Pdx1*^*Cre*^*;Vglut2*^*fx/fx*^ mice demonstrate changes in spectral power that align directionally with those observed in the *Ptf1a*^*Cre*^*;Vglut2*^*fx/fx*^ mice, reinforcing the robustness of the impact of cerebellar DBS in disease circuits across genetic models of dystonia.

Despite our incomplete understanding of the mechanisms through which cerebellar stimulation alleviates motor or non-motor symptoms [53,54], both the data presented in this study and our previous work [7,30,43] suggest that overriding abnormal cerebellar communication with DBS may be instrumental for its effectiveness. Additionally, sleep improvements observed with our FN ​+ ​INT-DBS may be mediated by the release of 5-hydroxyryptamine (serotonin). Indeed, it is known that electrical stimulation of the FN can modulate the release of serotonin in the frontal lobe of rats [39], and serotonin is known to play a complex role in sleep, affecting wakefulness, REM, and the ability to maintain sleep [55,56]. It has also been demonstrated that pharmacogenetic suppression of the 5HT-2A receptors in the FN is sufficient to mediate stress-induced dystonia in the *tottering* mouse [57]. Although an extensive analysis of the molecular mechanisms through which stimulation of the FN mediates improvements in sleep were outside the scope of this study, the current data do provide a fruitful avenue for future research to improve or fine-tune therapeutic outcomes by considering both the molecular and circuit properties.

Therapeutic outcomes could be improved further by making DBS more precise in targeting specific cerebellocortical pathways that would optimize its benefits but minimize other unwanted side effects. The combined evidence from our histological examination and the post-stimulation behavioral outcomes—namely, the reduction of motor dysfunction and sleep disturbances during FN ​+ ​INT-DBS—indicates the successful influence of both nuclei. Accordingly, the position of our stimulation electrodes was selected to capitalize on the distance between these nuclei. Given the conductive nature of the white matter, we hypothesize that our electrical stimulation effectively reached both nuclei. However, we also recognize that by targeting the white matter situated between these nuclei, we are likely to also influence several cerebellar afferent and efferent pathways. This encompasses the mossy and climbing fiber inputs to the cerebellum, as well as internal axons passing over the cerebellar nuclei. Regardless, the potentially broad impact of DBS in this region could indeed be beneficial, aligning with our goal of modulating diverse behavioral modalities to address both sleep and motor deficits in our mouse models.

Reduction in EMG power during REM sleep with stimulation is particularly striking, given that muscle atonia, typically a defining characteristic of this sleep state, was not visible in the *Ptf1a*^*Cre*^*;Vglut2*^*fx/fx*^ mice without the aid of cerebellar DBS. This finding indicates that both DBS paradigms (FN ​+ ​INT and INT only) can address motor dysfunction across different arousal states, including sleep. However, improvement in motor function alone, as observed with INT-DBS, could not overcome the sleep deficits associated with dystonic mice (Fig. 5a–g). This corroborates our previous finding of sleep disruptions in dystonic mice depending on cerebellar dysfunction but not motor symptoms in our mutant mice [16]. Similarly, improvement in sleep with FN ​+ ​INT-DBS did not prevent the re-emergence of baseline high-amplitude EMG activity on the next day (fourth recording day, DBS-OFF). This suggests that reducing the dystonia-associated sleep changes is insufficient, at least in the short-term, for overcoming motor dysfunction in our dystonic mice.

It is provocative to consider that the improvements in sleep in our studies could be enhanced by expanding our cerebellar DBS target from the INT to include more of the FN. However, the FN also has extensive connections to diverse autonomic, cognitive, and affective functions [58,59] that would likely result in additional and potentially less desirable effects. Electrical stimulation of the FN has been shown to evoke cardiovascular responses, predatory attacks, grooming, and consummatory behaviors [60,61]. In humans, electrical stimulation around the FN during surgery has resulted in respiratory tachypnea [5]. For this reason, direct targeting of the FN for dystonia would have to proceed with caution. Moreover, a better understanding of the cellular identities, molecular composition, and internal circuitry of the FN is much needed. Future improvements in cerebellar DBS targeting could involve using electrodes with densely packed stimulation contacts that can be independently activated to manipulate the electric field toward specific pathways of interest to optimize the benefits while avoiding unwanted effects [[62], [63], [64]].

Collectively, our findings highlight the promising therapeutic potential of FN ​+ ​INT-DBS in dystonia, demonstrating its capacity to simultaneously reduce both the motor and non-motor symptoms that can exacerbate one another. This is particularly noteworthy as the importance of addressing sleep dysfunction is increasingly recognized, not only in dystonia but in diverse societal contexts, given its substantial impact on quality of life, propensity to lead to various comorbidities, and association with impaired motor function and learning. Our results further reinforce the central role of the cerebellum within the nervous system's broader neural networks, and consequently, the profound potential of cerebellar-targeted interventions across diverse symptoms. The wide-reaching impact of cerebellar dysfunction, and the correspondingly widespread implications of its modulation through DBS, underscore the necessity and promise of cerebellar interventions in the context of motor disease and beyond. Therefore, our FN ​+ ​INT-DBS paradigm stands to serve as a comprehensive potential platform and model for addressing the spectrum of dystonia symptoms.

## Ethical approval

Animal experimentation: All animals (mice) were housed in an AALAS-certified facility that operates on a 14-h light cycle. Husbandry, housing, euthanasia, and experimental guidelines were reviewed and approved by the Institutional Animal Care and Use Committee (IACUC) of Baylor College of Medicine (protocol number: AN-5996).

## Data availability

All data generated or analyzed in this study are included in the manuscript and supporting files.

## Author contributions

Technical and conceptual ideas in this work were conceived by LESL and RVS. LESL and LHK performed experiments. LESL, LHK, and RVS performed data analysis and data interpretation. LESL, LHK and RVS wrote the manuscript. LESL, LHK, and RVS edited the manuscript.

## Declaration of competing interest

The authors declare the following financial interests/personal relationships which may be considered as potential competing interests: Roy V. Sillitoe reports financial support, administrative support, article publishing charges, equipment, drugs, or supplies, and travel were provided by The Hamill Foundation. Roy V. Sillitoe reports financial support, administrative support, article publishing charges, equipment, drugs, or supplies, and travel were provided by National Institute of Neurological Disorders and Stroke. Roy V. Sillitoe reports financial support, administrative support, article publishing charges, equipment, drugs, or supplies, and travel were provided by Dystonia Medical Research Foundation. Roy V. Sillitoe reports financial support, administrative support, article publishing charges, equipment, drugs, or supplies, and travel were provided by National Institute of Child Health and Human Development. Linda H. Kim reports financial support, article publishing charges, equipment, drugs, or supplies, and travel were provided by Dystonia Medical Research Foundation. Roy V. Sillitoe reports a relationship with Raynor Cerebellum Foundation that includes: board membership. Roy V. Sillitoe serves on the Board of Reviewing Editors at eLife. If there are other authors, they declare that they have no known competing financial interests or personal relationships that could have appeared to influence the work reported in this paper.
